# How Much Is Enough: A Randomized Non-Inferiority Trial Comparing Three Bodyweight Training Protocols

**DOI:** 10.3390/jfmk11020240

**Published:** 2026-06-17

**Authors:** Joshua J. Aube, Peter J. Mendolia, Kristi L. Storoschuk, Ely Wyman, Mason D. Peberdy, John J. Wu, Nia Simpson-Stairs, Paul A. Swinton, Brendon J. Gurd

**Affiliations:** 1 School of Kinesiology and Health Studies, Queen’s University, Kingston, ON K7L 2N9, Canada; 2 School of Health, Robert Gordon University, Aberdeen AB10 7QE, UK

**Keywords:** bodyweight training, cardiorespiratory fitness, exercise volume, high-intensity interval training

## Abstract

**Purpose:** The current study tested the hypothesis that a bodyweight training (BWT) protocol with a higher weekly time commitment and training volume would produce greater improvements in cardiorespiratory fitness compared to lower time commitment/volume BWT protocols. **Methods:** Fifty-eight (*n* = 21 males; *n* = 37 females) recreationally active, healthy, young adults were randomized to either a 16 (TAB, *n* = 20), 27 (5BX, *n* = 19), or 90 min/week (AMRAP, *n* = 19) BWT protocol for 6 weeks. Peak work rate (WRpeak) was measured pre- and post-intervention using a cycle ergometer graded exercise test. VO_2_peak was estimated (eVO_2_peak) using a simple linear regression of WRpeak and VO_2_peak generated from a previous study (*n* = 26; 13M/13F). **Results:** TAB and 5BX yielded non-inferior improvements in eVO_2_peak. The 95% confidence intervals of the mean difference in change did not cross our non-inferiority margin of −2.6 mL/kg/min (TAB—AMRAP = 95% CI = −1.16 to 2.07; 5BX—AMRAP 95% CI = −0.29 to 2.89). **Conclusions:** Our results suggest that 16 and 27 min/week of minimalist BWT yields non-inferior improvements in CRF compared to 90 min/week and provides evidence supporting the efficacy of low-volume BWT protocols compared to higher volume protocols. The study was registered on Open Science Framework on 29 May 2023 (OSF registration).

## 1. Introduction

Despite the known benefits of physical exercise, physical inactivity remains a global problem [1,2,3]. A lack of time, space, and money are among the most commonly reported barriers to regular physical activity [4,5,6]. A minimalist approach to physical activity–exercise that requires minimal time, space, and cost—may mitigate the most prevalent barriers to regular physical activity and thus reduce rates of physical inactivity.

High-intensity interval training (HIIT) utilizes bouts of near-maximal exertion interspersed with brief rest intervals. HIIT is a time-efficient means of improving cardiorespiratory fitness (CRF) [7]—an important indicator of quality of life [8], disease risk [9], and all-cause mortality [10,11]. Bodyweight training (BWT) is a minimalist variant of HIIT that maintains the same time-efficiency and efficacy for improving CRF as traditional HIIT [12,13], while also eliciting improvements in muscular strength and endurance [14,15,16,17]. Although numerous different BWT protocols improve CRF and muscular strength and endurance, they vary in weekly training volume, training time, work-to-rest ratio, exercise selection, and training intensity. Given that time is an often-cited barrier to completing physical activity, understanding the minimal dose of BWT associated with improved CRF is important. However, to the best of our knowledge, a comparison of BWT protocols differing in weekly training time/volume does not exist.

Thus, the present study aimed to perform a non-inferiority trial to assess whether low-volume BWT protocols induce inferior improvements in CRF compared to higher volume BWT protocols. Specifically, we compared changes in CRF following three previously published protocols known to improve CRF with weekly training time commitments of 16 [14], 27 [17], and 90 [15] min. The primary purpose was to assess this question using estimated peak oxygen uptake (eVO_2_peak) at a between-group difference of 2.6 mL/kg/min. This value was set as the non-inferiority margin for this trial because: (1) a 1-MET (3.5 mL/kg/min) higher CRF is associated with reduced risk of mortality and cardiovascular disease [10], (2) small improvements in CRF across time are associated with reduced risk of mortality and disease [18,19], (3) it aligns with the mean changes in CRF observed in previous BWT studies [14,15,17], and (4) it allowed a detectable difference between groups at a feasible sample size (small/medium effect size; *n* = 51). Based on the established relationship between training volume and improvements in CRF [20,21] following traditional exercise training we hypothesized BWT to improve CRF in a volume-dependent manner. Specifically, we expected 16 and 27 min/week of BWT would yield inferior improvements compared to 90 min/week. The secondary purpose was to examine the relationship between weekly BWT volume and changes in muscular strength and endurance. Similar to CRF, we hypothesized greater improvements in muscular strength and endurance with increasing BWT volume.

Studies comparing response rates across different exercise intensities, amounts, or frequencies provide consistent evidence that higher volumes of aerobic exercise are associated with greater response rates [22,23,24]. Much less is known about the impact of resistance exercise volume on response rate. To our knowledge only two studies have compared response rates following different volumes of resistance training [25,26]. Although limited, data from these studies suggest that the impact of augmented training volume on individual response rates may vary depending on the protocol prescribed. However, given the established relationships between response rate and mean change [22] and resistance training volume and strength gains [27], we hypothesized individual response rate would increase with increasing BWT volume. To test this hypothesis, we performed an exploratory Bayesian analysis with expert prior elicitation.

## 2. Methods

### 2.1. Participants

Participants were included if they met the following criteria: (1) between the ages of 18 and 40, (2) non-smokers, (3) no previous history of cardiometabolic disease, (4) no involvement in a structured exercise training program in the past 6 months, (5) not currently taking any medications aside from contraceptives and selective serotonin reuptake inhibitors, and (6) met the criteria for being classified as recreationally active [28]. The body-mass index (BMI) requirement of <30 kg/m^2^ from our trial registration was removed within the initial eight weeks of participant recruitment due to difficulties recruiting. Physical activity levels and readiness were assessed using a 7-day Physical Activity Readiness Questionnaire for Everyone (PAR-Q+). The study received research approval from the Health Sciences Research Ethics board at Queen’s University (#6038541). The study was registered on Open Science Framework on 29 May 2023 (OSF registration, https://osf.io/u37hb/overview?view_only=c0140f22b0924765a874190108162d7e). All participants provided signed informed consent prior to participation.

### 2.2. Experimental Design

**Figure 1:**
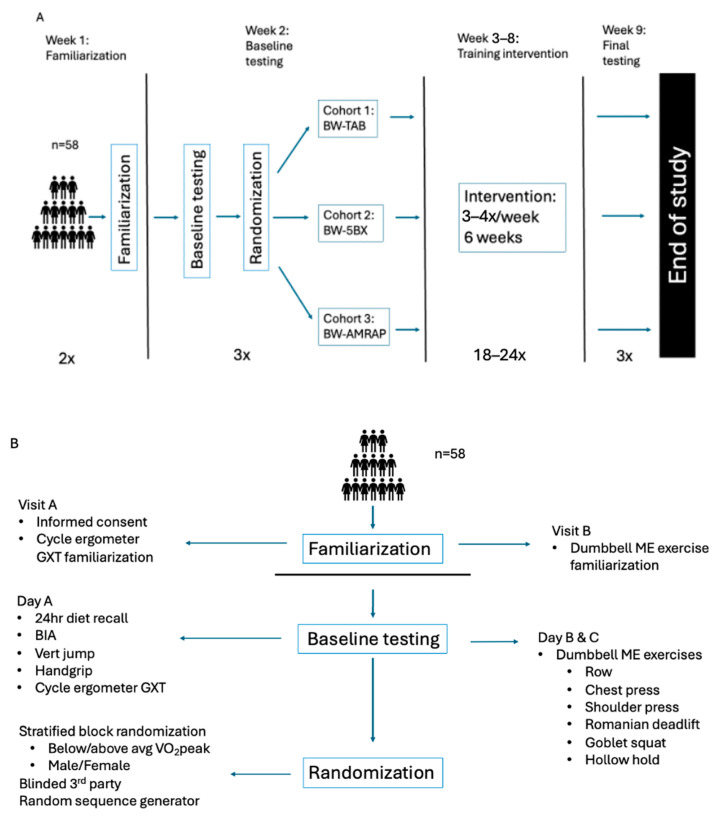
Study design and participant flow. (**A**) Overview of the 9-week study protocol. Following familiarization and baseline testing, participants (*n* = 58) were randomized to one of three bodyweight training interventions (TAB, 5BX, or AMRAP) and completed 6 weeks of training (3–4 sessions·week^−1^) before final testing. (**B**) Detailed timeline of study visits, including familiarization procedures, baseline assessments, and randomization.

A randomized, parallel arm design was used to examine the effect of BWT volume on CRF (see Figure 1. for flowchart depicting study design). Minor modifications were made to the three previously validated BWT protocols to ensure all protocols could be performed indoors in minimal space and without weights or equipment. Participant recruitment and data collection took place between June 2023 and May 2024 in Kingston, Ontario, Canada. All participants completed familiarization and baseline testing prior to randomization into one of three 6-week exercise interventions. All visits were supervised by a researcher. Verbal encouragement was provided during every visit with no differences between groups. Following completion of their respective training intervention all participants completed post-intervention testing. Baseline testing was completed ~48–72 h before each participant’s first training session and post-intervention testing was completed in an identical fashion to baseline testing ~48–72 h after the final training session. None of the exercises included in pre/post-testing were utilized during the training interventions to mitigate an effect of learning on outcome assessment.

The current study implemented randomized sequence generation, allocation concealment, outcome assessor blinding, and trial registration (OSF registration) to mitigate selection, performance, observer, and outcome reporting biases as we have done previously [29]. Participants were randomized following the final day of baseline testing using stratified block randomization. Males and females were stratified by sex before being divided into two blocks based on their respective CRF at baseline—above-average (>50 mL/kg/min for males, >45 mL/kg/min for females) or below-average (<50 mL/kg/min for males, <45 mL/kg/min for females). Within each of the four blocks, participants were randomized into 1 of 3 BWT protocols by a blinded third party using a random sequence generator. The purpose of this approach was to ensure an equal dispersion of sex and baseline fitness within each of the three experimental groups. Statistical analysis was performed by an investigator blinded to group allocation to mitigate detection bias. Participants were not informed of the primary hypothesis of the current study to mitigate performance bias. Full details of methods used to mitigate the influence of experimental bias are presented in Supplemental File S1.

### 2.3. Familiarization

Familiarization was conducted over two consecutive days: familiarization A and B. Familiarization A consisted of informed consent, assessment of inclusion criteria (PAR-Q+, 7-day physical activity recall, 6-month training recall), and a WRpeak test familiarization (as described below). All required exercises for each of the three interventions were demonstrated by a student investigator. Participants had the opportunity to lightly practice any exercises to help facilitate learning.

Familiarization B consisted of vertical jump and muscular endurance testing. ~48–72 h rest was given between familiarization B and the first day of baseline testing. See OSF protocol for full details of all testing completed during familiarization visits (OSF registration). Details of familiarization and physiological testing are also presented in Supplemental File S2 (Table S1).

### 2.4. Physiological Testing

Participants were instructed not to change dietary habits or weekly physical activity throughout the study and not to consume alcohol or caffeine 24 h prior to any testing protocol. Participants recorded a 24 h dietary recall and 7-day physical activity recall prior to the first day of baseline testing, referred to their completed document on the final intervention day and asked to keep their diet and physical activity identical prior to post-testing. Baseline and post-intervention testing were conducted in an identical fashion over three different visits; testing day A, B, and C. Testing day A consisted of a 24 h dietary recall, 7-day physical activity recall, body composition, vertical jump, isometric handgrip strength, and WRpeak test (See OSF protocol for full details). Due to an error in data collection the vertical jump data could not be analyzed and thus, was not included in the manuscript.

Briefly, the WRpeak incremental protocol consisted of a 5 min load-less warmup followed by a step increase to 80 Watts (W) for 1 min and subsequent increases of 24 W/min until volitional fatigue (determined by the inability of the participant to maintain a cadence of 60 revolutions per minute (RPM)). RPM was collected continuously throughout the test after the initial 5 min warmup. WRpeak was calculated using the RPMs recorded during the final 30 s of exercise and is reported as an index of absolute CRF (W) throughout the remainder of this manuscript. WRpeak tests were completed on a stationary cycle ergometer (Monark, Ergomedic 874E Varberg, Sweden).

Changes in relative VO_2_peak (mL/kg/min) were estimated (eVO_2_peak) from WRpeak derived from the incremental cycle ergometer test described above, and a regression equation from a separate sample generated using the same cycle ergometer and incremental test protocol. The previous sample (*n* = 26; 13F/13M) exhibited similar characteristics as the population studied in the current study (F, 22 ± 3 yr, BMI = 23 ± 2, WRpeak = 237 ± 23 W, VO_2_peak = 42 ± 5 mL/kg/min; M, 22 ± 2, BMI = 24 ± 2 kg/m^2^, WRpeak = 285 ± 40 W, VO_2_peak = 47 ± 4 mL/kg/min). The final prediction equation for eVO_2_peak (mL/min) was VO_2_ (mL/min) = 12.3 × WRpeak (W) + 250 (r^2^ = 0.81, SEE = 315 mL). The resulting eVO_2_peak value was divided by participant bodyweight to derive relative CRF (mL/kg/min).

Testing day B and C consisted of upper and lower body muscular endurance testing, respectively. Muscular endurance was tested by measuring the maximal number of repetitions in a single working set for each of dumbbell row, chest press, and shoulder press, Romanian deadlift (RDL), goblet squat, and hollow hold. Participants completed one warmup set for each movement to prepare the nervous system, acutely reacquaint participants with the unique movement patterns of each exercise, increase blood flow to the working muscles and joints, and to do so with minimal fatigue accumulation. The warmup set was 65% of the working weight for each movement, rounded to the nearest 10. Detailed protocols for muscular endurance testing can be found in the OSF protocol (OSF registration) and in Supplemental File S2 (Table S1).

### 2.5. Interventions

Warmup and cooldown for all training sessions were identical for all three groups. The warmup consisted of 15 s each of 4 dynamic stretches; front leg swings, lateral leg swings, lateral arm swings, and arm circles. The cooldown consisted of 1 min of walking in place. All participants wore an Apple Watch (Series 3, Cupertino, CA, USA) or FitBit (Versa Lite, San Francisco, CA, USA) to track intrasession average and peak heart rate (HR). Exercise and/or circuit repetitions were also recorded for each workout to track performance and characterize each protocol.

### 2.6. BWT: TAB

TAB (16 min/week) followed a modified version of a previously described Tabata bodyweight protocol [14] M (see Supplemental File S2 (Table S2) for details). Participants exercised 4 days/week. The only modification made was to perform the squat thrusts without weight. On each training day participants performed 8 intervals of 20 s all-out intensity interspersed with 10 s of rest of one of four exercises; burpees, mountain climbers, jumping jacks, or squat thrust to shoulder press. Participants were encouraged to perform as many repetitions per interval as possible while maintaining correct form.

### 2.7. BWT: 5BX

5BX (27 min/week) followed a modified version of a previously described “5 Basic Exercises” (5BX) protocol [17] (see Supplemental File S2 (Table S3) for details). Participants exercised 3 days/week. Participants performed 5 intervals of 60 s all-out intensity interspersed with 60 s of rest. On each training day participants performed the same circuit consisting of one interval of burpees, high knees, split squat jumps, mountain climbers, and squat jumps, respectively. Participants were encouraged to perform as many repetitions per interval as possible while maintaining correct form.

### 2.8. BWT: AMRAP

AMRAP (90 min/week) followed a modified version of a previously described bodyweight protocol [15] (see Supplemental File S2 (Table S4) for details). Participants exercised 3 days/week. Participants performed as many repetitions of the circuit as possible in 30 min. Participants were instructed to take rest breaks as needed but were encouraged to take as few as possible. The only modifications from the original protocol were to repeat the 5th circuit again in week 6, and to substitute the 200 m sprints with high knees (10/leg). This is the only group with predetermined repetitions per exercise and without predetermined rest breaks.

### 2.9. Statistical Analysis

An a priori sample size calculation was performed for the primary outcome: group x time interaction for eVO_2_peak determined using a 2-way repeated measures ANOVA. G*Power (version 3.1.9.7) revealed that a sample size of 51 was required to detect a small/medium effect size (f = 0.175, *α* error probability = 0.05, 1 − β error probability = 0.8, correlation r = 0.7). This effect size estimate corresponds to a difference in eVO_2_peak between groups of 2.6 mL/kg/min, a clinically meaningful change in CRF [18,19] that was informed by the absolute change in VO_2_peak (and standard deviation, SD) observed [14,15,17], and feasibility considerations related to the total number of participants we believed we could train in a reasonable timeframe. A summary of the data used to estimate our anticipated sample size is presented in Supplemental File S2 (Table S5).

Non-inferiority tests for improvement in eVO_2_peak and WRpeak were also performed. Non-inferiority tests were conducted by constructing a 95% confidence interval for the mean difference in eVO_2_peak improvement between TAB/5BX and AMRAP. Specifically, the mean change in AMRAP was subtracted from the mean change in TAB/5BX (i.e., TAB—AMRAP and 5BX—AMRAP) and 95% confidence intervals were calculated using GraphPad Prism version 8.0 (GraphPad Software, San Diego, CA, USA). Non-inferiority was declared if the 95% confidence intervals of the mean difference in eVO_2_peak improvements did not cross the non-inferiority margin. The a priori non-inferiority margin of −2.6 mL/kg/min was used for the reasons discussed above. The same analysis was performed for WRpeak using a non-inferiority margin of −14 W (the mean change in W associated with a −2.6 mL/kg/min change in eVO_2_peak in our sample). Differences in the changes in eVO_2_peak and WRpeak were also compared using one-way ANOVAs.

Secondary outcomes for this trial (changes in muscular strength and endurance) were assessed using separate 2-way repeated measures ANOVAs (training group [TAB vs. 5BX vs. AMRAP]) × time (pre vs. post). An exploratory sex analysis was conducted for changes in eVO_2_peak using a 2-way ANOVA (sex x training group). One-way ANOVAs were used to examine between-group differences in HR (average and peak), and cardiovascular load (session and weekly). Significant interactions and main effects were analyzed using Tukey post hoc analysis. Corresponding effect sizes for ANOVAs were calculated and interpreted using partial eta squared (η^2^) values (small = 0.0099; medium = 0.0588; large = 0.1379) [30]. All statistical analyses were performed using GraphPad Prism version 8.0 (GraphPad Software, San Diego, CA, USA). Effect sizes for within- and between-group post hoc comparisons were calculated using Hedge’s G for a between-subjects design and were interpreted as small (Hg = 0.2), medium (Hg = 0.5), and large (Hg = 0.8) [30]. All analyses followed a per-protocol approach and no intention-to-treat or missing data sensitivity analysis was performed. Statistical significance was accepted at *p* < 0.05, and all data are presented as means ± SD.

An exploratory analysis (effect of BWT volume on individual response rates) was conducted with a Bayesian approach and expert prior elicitation. We conducted a structured expert elicitation with four international experts using the quartile method within the MATCH/SHELF framework [31]. For each outcome, experts provided their beliefs about population mean change for the reference intervention (TAB) and then about pairwise mean differences (5BX—TAB and AMRAP—TAB). Expert beliefs about within-group variability were used as residual standard deviation priors. After each set of quartiles, we used MATCH to fit a provisional distribution and immediately fed back a graphical density together with an implied central probability statement (for example, “there is ~80% probability the true mean lies between X and Y”). Experts were invited to revise until the display matched their belief. Full details of the elicitation process are presented in Supplemental File S1.

After elicitation, we fit separate Gaussian intercept–delta models for each outcome, with TAB as the reference level and 5BX/AMRAP encoded as deltas. Models were fit in brms [32] (4 chains: 4000 iterations per chain with 2000 warmup) and convergence/precision were verified using standard diagnostics (all $\hat{R}\;\leq \;1.01$, adequate bulk/tail ESS). To estimate responder proportions by group, we drew posterior predictive samples for new observations at the group means and, for each draw, computed the fraction exceeding prespecified, exercise science-specific distribution-based change thresholds defined from the baseline sample SD (Small: 0.11 × SD; Medium: 0.38 × SD; Large: 0.67 × SD) [33]. We summarized these draw-wise proportions within each group by the posterior median and 95% credible interval (2.5th, 97.5th percentiles) (Supplemental Figure S5).

Anticipating strong correlations among muscular endurance outcomes, we fit a single stacked hierarchical intercept–delta model that partially pools information across tests while allowing outcome-specific effects. The random-effects structure comprised (i) a random intercept for outcome (outcome-specific baseline under TAB), (ii) random slopes of intervention by outcome for 5BX and AMRAP (specified uncorrelated for stability), and (iii) a subject random intercept to accommodate multiple outcomes per participant. Posterior predictive draws from this hierarchical model were again used to compute responder proportions by group for new individuals, integrating subject-level variation and integrating the underlying structure across outcomes.

## 3. Results

### 3.1. Participant Characteristics

**Figure 2:**
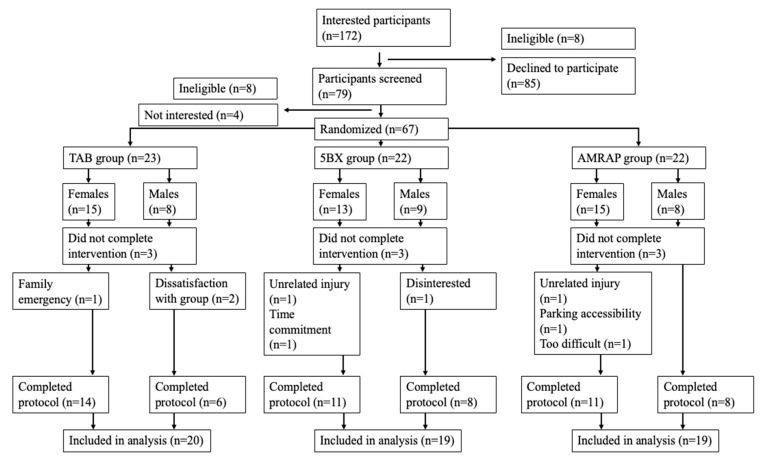
CONSORT flow diagram of participant recruitment, allocation, follow-up, and analysis.

A total of 79 individuals were screened for participation and 58 completed all aspects of the study (see Figure 2 for flowchart detailing recruitment, screening, exclusion, randomization, dropouts and completion, and Table 1 for participant baseline characteristics). Session attendance was high for all groups (TAB, 90 ± 7%; 5BX, 87 ± 7%; AMRAP, 89 ± 6%)—all participants completed at least 75% of prescribed sessions.

### 3.2. Characterization of BWT Protocols

A significant group effect was observed for average training HR (Supplemental Figure S1A; *p* < 0.001; η^2^ = 0.33), peak HR (Supplemental Figure S1B; *p* < 0.001; η^2^ = 0.30), and cardiovascular load (session volume, Supplemental Figure S1C, *p* < 0.001, η^2^ = 0.99; weekly training volume, Supplemental Figure S1D, *p* < 0.001, η^2^ = 0.99). Post hoc tests revealed AMRAP was greater than TAB for all three variables (Supplemental File S1). Average total repetitions per week (TAB and 5BX) and circuits per session (AMRAP) are presented in Supplemental Figure S2.

### 3.3. Primary Analysis

**Figure 3:**
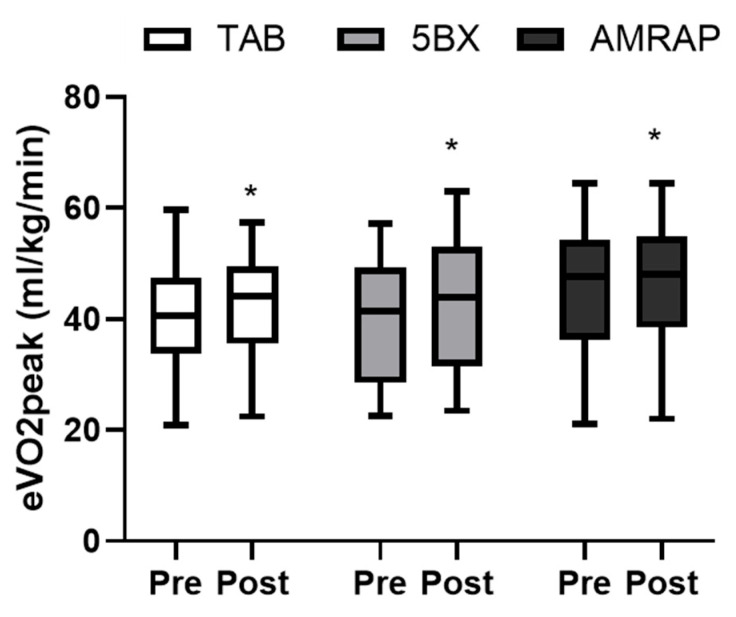
Boxplots showing eVO_2_peak at baseline (Pre) and following 6 weeks of BWT (Post) for TAB, 5BX and AMRAP groups. * Significant (*p* < 0.05) effect of time.

Two-way ANOVA (group x time) analysis revealed a significant effect of time (*p* < 0.01; η^2^ = 0.85) but no significant interaction (*p* = 0.31; η^2^ = 0.06) or effect of group (*p* = 0.36; η^2^ = 0.08) for eVO_2_peak (Figure 3). Similarly, two-way ANOVA (group x time) analysis revealed a significant effect of time (*p* < 0.001; η^2^ = 0.39) but no significant interaction (*p* = 0.42; η^2^ = 0.03) or effect of group (*p* = 0.09; η^2^ = 0.08) for absolute WRpeak (Supplemental Figure S3).

**Figure 4:**
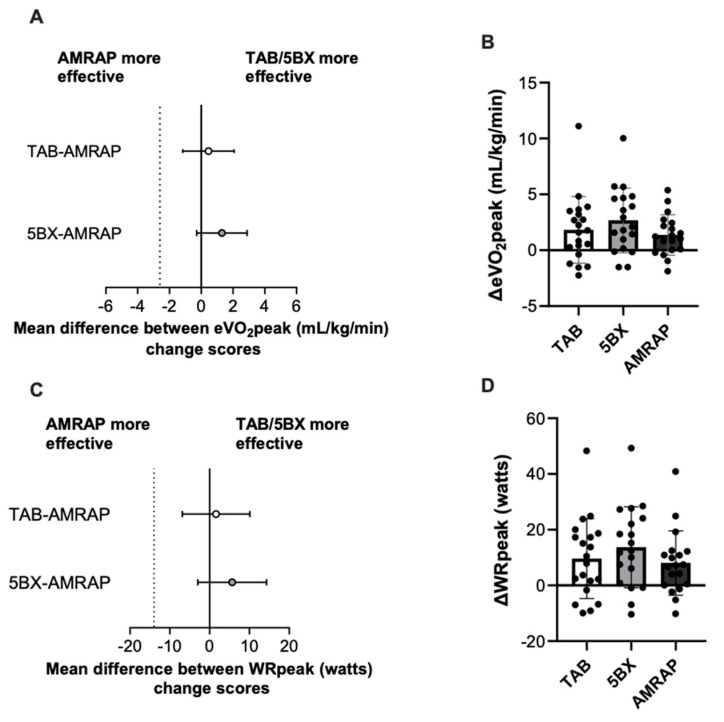
(**A**) An estimation plot, showing the point estimate and 95% confidence interval, is presented for the difference in training-induced eVO_2_peak change scores between TAB/5BX and AMRAP, with the dashed line representing the a priori specified non-inferiority margin of −2.6 mL/kg/min. (**B**) Average and individual changes in eVO_2_peak following training. (**C**) An estimation plot, showing the point estimate and 95% confidence interval, is presented for the difference in training-induced WRpeak change scores between TAB/5BX and AMRAP, with the dashed line representing the corresponding non-inferiority margin of −14 W. (**D**) Average and individual changes in relative WRpeak following training.

The bounds of the 95% confidence intervals for the mean difference in change in eVO_2_peak (TAB vs. AMRAP −1.2 to 2.1 mL/kg/min; 5BX vs. AMRAP −0.3 to 2.9 mL/kg/min) did not include the a priori determined non-inferiority margin of −2.6 mL/kg/min (Figure 4A). Further, the difference in the improvement in eVO_2_peak after training was not significantly different between groups (Figure 4B; *p* = 0.31, η^2^ = 0.04). The bounds of the 95% confidence intervals for the mean difference in change in WRpeak (TAB vs. AMRAP −6.8 to 10.1 W; 5BX vs. AMRAP −2.9 to 14.3 W) did not include the corresponding non-inferiority margin of −14 W (Figure 4C). The impact of training on WRpeak was not significantly different between groups (Figure 4D; *p* = 0.31, η^2^ = 0.04).

Exploratory analysis of the effect of sex on changes in eVO_2_peak are presented in Supplemental Figure S4. Additionally, two-way ANOVA analysis revealed no effect of sex (*p* = 0.20; η^2^ = 0.03), group (*p* = 0.38; η^2^ = 0.04), or interaction (*p* = 0.45; η^2^ = 0.03).

### 3.4. Secondary Analysis: Muscular Strength and Endurance

A series of repeated measures ANOVAs revealed no interaction effects (*p* > 0.05, η^2^_p_ < 0.08) or main effects of group (*p* > 0.05, η^2^_p_ < 0.08) across all variables (Table 2). Significant main effects of time were detected (*p* < 0.005, η^2^_p_ > 0.14) across all variables (Table 2).

### 3.5. Exploratory Individual Response Analysis

Using informative priors, we estimated posterior distributions for mean change scores by group and outcome, shown alongside the corresponding prior and empirical (sample) distributions (Supplemental Figure S5). Across outcomes, the priors exerted a modest influence: posterior means moved slightly toward elicited values and posterior spreads contracted relative to sample-only estimates, consistent with partial pooling. The elicited ordering from experts followed the hypothesized effect of volume on training adaptation (TAB < 5BX < AMRAP). This order was generally reflected in the posterior distributions where AMRAP tended to demonstrate larger improvements than TAB (Supplemental Figure S5). However, for several outcomes the posterior distributions for TAB and 5BX showed substantial overlap (indicating uncertain separation).

**Figure 5:**
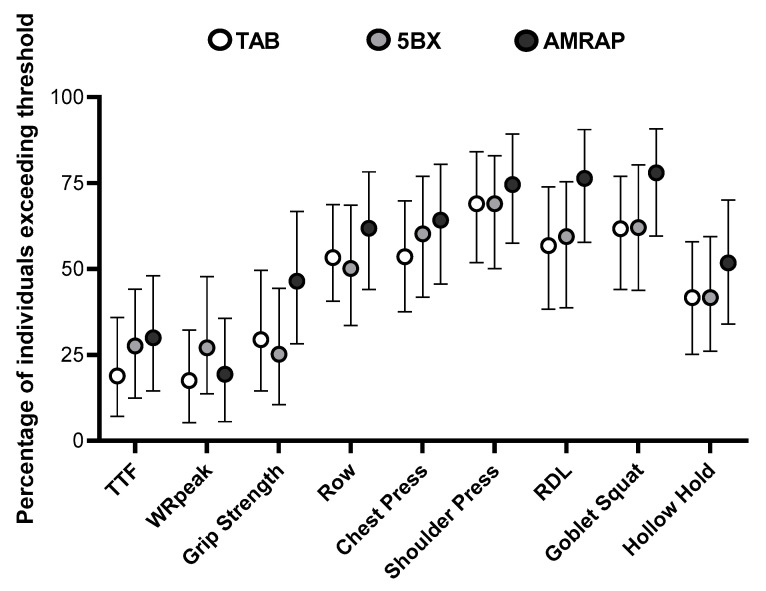
Responder rates using a medium effect-size threshold expected across interventions and outcomes. Symbols are posterior point estimates of the percentage of positive responders. Lines represent 95% credible intervals.

Posterior responder rates from the outcome-specific models at small (0.11·SD), medium (0.38·SD), and large (0.67·SD) thresholds are presented in Supplemental Figure S6. At the small threshold, responder rates typically lay around 60–75%. At the large threshold, rates fell to <10% for TTF, WRpeak, and handgrip strength, whereas several muscular endurance outcomes remained higher (about 25–50%+) (Supplemental Figure S6). For ease of viewing, only outcome-specific models for medium effect thresholds are presented in Figure 5. Point estimates suggested an ordered effect of volume for time to fatigue and many muscular endurance tests, with clearer increases in response rate for AMRAP vs. TAB than for 5BX vs. TAB. However, point estimates for WRpeak suggest a greater response rate in the 5BX group with uncertain separation between AMRAP and TAB.

Motivated by these distinct patterns and the anticipated correlations among muscle endurance outcomes, we fitted the hierarchical stacked model. The pooled fixed-effect estimates supported an overall effect of volume: TAB mean change = +5.7 repetitions (95% CrI: 1.9, 9.2); 5BX−TAB = +1.2 (95% CrI: −1.2, 3.6); and AMRAP−TAB = +3.7 (95% CrI: 0.5, 6.8). This indicated the clearest improvement for AMRAP, with considerable overlap between TAB and 5BX.

## 4. Discussion

The current study tested the hypothesis that TAB and 5BX would yield inferior eVO_2_peak improvements compared to AMRAP. Contrary to our hypothesis, repeated measures ANOVAs revealed no interaction for improvements in eVO_2_peak (or WRpeak) between BWT protocols indicating that all three protocols elicited similar improvements. Further, non-inferiority analysis confirmed that TAB and 5BX produced non-inferior improvements to AMRAP for eVO_2_peak and WRpeak. These equivalent responses occurred despite significant differences in total training time (min/week) and volume (i.e., cardiovascular load; Supplemental Figure S1). We also demonstrated similar improvements in muscular strength and endurance between all three protocols (Table 2). Interestingly, exploratory Bayesian analyses produced evidence of an ordered effect of BWT volume on individual response rates across all variables, except WRpeak, with the clearest difference between AMRAP and TAB (Figure 5). A subsequent analysis using a hierarchical stacked model suggested AMRAP may induce larger improvements in muscle strength and endurance than TAB.

Our results suggest that, of the BWT protocols investigated, TAB is the most time efficient means of improving eVO_2_peak. Conversely, our individual response analysis suggests that higher volume BWT protocols may be important for those targeting improvements in muscle strength and endurance.

### 4.1. Training-Induced Improvements in Cardiorespiratory Fitness

Our demonstration of non-inferior improvements in eVO_2_peak following low-volume BWT are consistent with several previous studies where multiple BWT protocols differing in volume were compared [34,35,36]. For example, doubling BWT volume failed to augment CRF improvements in recreationally active adults [34,36]. Further, increasing BWT frequency from once per day (36 min/week) to twice per day (72 min/week) had no effect on CRF in young, untrained adults [35]. Both the current study, and past studies, have a limited training duration (4–6 weeks), a factor that could confound our results given the known impact of BWT duration on CRF [37]. However, the evidence currently available suggests that high volumes of BWT are not required to improve CRF.

An important caveat for the current study is the difference in work-to-rest ratio between our protocols. Although, all three protocols prescribed “all-out” intensity, the AMRAP protocol employed self-prescribed rest intervals (participants were instructed to use rest intervals as needed but as infrequently as possible). Work-to-rest intervals of HIIT protocols can have a significant impact on workout intensity [38] and thus differences in prescription may have influenced the adaptive stimulus associated with each protocol. Although HR data would suggest the AMRAP protocol elicited similar cardiovascular stress (Supplemental Figure S1) it is possible that the non-inferior effects of TAB/5BX compared to AMRAP resulted from a combination of differences in volume and intensity.

### 4.2. Training-Induced Improvements in Muscular Strength and Endurance

Similar to CRF, we observed equivocal improvements in muscle strength/endurance across all three BWT protocols. Typically, greater volumes of resistance training lead to greater strength gains [27]. However, the BWT protocols in the present study are more similar to HIIT than traditional resistance training (very low weight, high repetitions, work intervals done at an “all-out” intensity, brief rest periods). We speculate that improvements in oxidative capacity typically observed following HIIT [39,40] contributed to improved muscle endurance. If the mechanisms responsible for improvements in muscular endurance and CRF following BWT are similar, it is thus unsurprising the effect of BWT volume on muscular strength and endurance mirrors that of CRF.

### 4.3. Response Rate Analysis

Our exploratory Bayesian analysis found evidence of an ordered effect of training time/volume on response rates for muscular strength and endurance with the largest difference observed between TAB and AMRAP (Figure 5). This result is contrary to our frequentist analyses (see Table 2) indicating a lack of difference in mean change—a major driver of response rate [22]. It is important to note that differences in response rates do not distinguish between different sources of variation [41] and our results should not be interpreted as evidence for reductions in response variability. Rather the greater response rates observed following AMRAP vs. TAB should be interpreted as initial evidence that higher time/volume BWT may improve an individual’s chances of experiencing a meaningful increase in muscle strength and endurance.

Our Bayesian analysis identified evidence of an ordered effect of BWT time/volume on mean change in muscular strength and endurance across pooled outcomes, with the clearest difference between TAB and AMRAP (AMRAP − TAB = +3.7 [95% CrI: 0.5, 6.8]). It is not uncommon for Bayesian analysis to produce different results from frequentist analysis. Although our priors were relatively flat (reflecting the uncertainty in expert elicited answers), they did exert a small effect of the posterior distribution (Supplemental Figure S5). Further the use of a hierarchical stacked model to pool muscular strength and endurance data added power to identify differences in mean changes between groups. Taken together, it is possible that AMRAP produced slightly larger improvements in muscular strength and endurance compared to TAB. However, this effect was small, and we were underpowered for frequentist analyses with our sample size.

## 5. Strengths and Limitations

Risk of bias is implicated as a cause of augmented treatment effect estimates [42] and the generation of irreproducible data [43]. Previous studies using the BWT protocol investigated in the current study did not report blinded outcome assessment or trial registration [14,15,17], and only one reported participant allocation concealment and an a priori sample size calculation [17]. Importantly, we were able to replicate the positive effects of BWT on CRF despite the rigorous experimental approach utilized in the current study (see Supplemental File S1).

A major limitation of the current work is the heterogeneity between BWT protocols. While the significant differences in weekly cardiovascular load are consistent with differences in weekly exercise volume (see Supplemental Figure S1D), the three BWT protocols examined also differ in training time, work-to-rest ratio, exercise selection, interval duration, and intensity distribution. These additional differences may have contributed to the results of our study, and we thus cannot comment with certainty on the direct impact of BWT volume on CRF.

The non-inferiority margin for CRF improvement in the current study was −2.6 mL/kg/min, a relatively large between-group difference, but the smallest detectable difference with a sample size that was feasible given financial and temporal constraints. It remains possible that small differences (<2.6 mL/kg/min) do exist between BWT protocols, but future studies with larger samples and statistical power are needed to test this possibility. Further, all statistical analysis followed a per-protocol approach and no intention-to-treat or missing data sensitivity analyses were performed. Given dropouts were balanced across groups (Figure 2), it is unlikely the per-protocol approach contributed meaningfully to the conclusions of our study.

The current study, along with previous studies examining the effects of BWT volume on CRF [34,35,36], is limited in intervention length (all studies 4–6 weeks in duration). It is possible that small differences between protocols could emerge following the training durations of >6 weeks. Further, given the potential for continued adaptation in CRF following longer exposure to training, especially following high-intensity/volume training [24], it remains unclear whether low-volume protocols would remain non-inferior following longer periods of training. Longer interventions with larger sample sizes are required to definitively determine whether low-volume BWT protocols are inferior to high-volume protocols.

## 6. Future Directions

Low-volume bodyweight training (BWT) may offer a practical strategy to improve CRF and muscle strength/endurance in clinical populations. Our demonstration that as little as ~16 min/week of BWT elicited non-inferior improvements in eVO_2_peak compared to higher volume protocols support the potential benefits of brief, accessible interventions in primary care, rehabilitation, and community health settings. Further, the potential for BWT to reduce common barriers to exercise (e.g., time, cost, equipment), could enhance implementation and patient adherence for home-based and telehealth delivery. However, the vast majority of BWT studies, including the current work, have studied young, healthy adults, and the acceptability of and efficacy of BWT in clinical populations remains unclear. Thus, there is a need for studies evaluating BWT protocols in clinical populations (e.g., cardiometabolic disease, older adults), over longer durations, within pragmatic and real-world settings.

## 7. Conclusions

We conclude that lower volume BWT protocols (TAB and 5BX) produce non-inferior changes in eVO_2_peak compared to a higher volume protocol (AMRAP). Our exploratory Bayesian analyses suggest AMRAP may produce larger changes in muscular strength and endurance compared to TAB; however, the effect was small and was not visible using a frequentist analysis with our sample size. Of the BWT protocols investigated in the current study, TAB appears to represent the most time-efficient BWT protocol for improving eVO_2_peak in a young, recreationally active population.

## Figures and Tables

**Table 1:** Participant characteristics.

		Age (Yr)	BMI (kg/m^2^)	WRpeak (W)	eVO_2_peak (mL/kg/min)
TAB	Females (*n* = 14)	23.9 (3.6)	23.9 (9.3)	179.5 (49.2)	36.8 (9.2)
	Males (*n* = 6)	21.3 (2.1)	21.8 (4.1)	227.2 (32.1)	40.2 (5.6)
5BX	Females (*n* = 11)	24.6 (6.1)	23.8 (3.7)	177.3 (53)	34.9 (10.1)
	Males (*n* = 8)	27.1 (7.9)	23.8 (3.7)	228.2 (50.2)	41.2 (10.7)
AMRAP	Females (*n* = 11)	22.9 (4.6)	23.8 (5.6)	214.7 (60.4)	42.1 (11.3)
	Males (*n* = 8)	23.7 (6.2)	24.7 (3.5)	260.2 (48.5)	43.7 (9.1)

Values presented as mean (standard deviation).

**Table 2:** ANOVA results.

	TAB (*n* = 20)			5BX (*n* = 19)			AMRAP (*n* = 19)			Effect Sizes
Measure	Pre	Post	△	Pre	Post	△	Pre	Post	△	
TTF (seconds)	608 (123)	624 (119)	16 (39)	641 (157)	677 (177)	36 (33)	705 (141)	738 (145)	32 (40)	T (*p* < 0.001, η^2^_p_ = 0.397) G (*p* = 0.080, η^2^_p_ = 0.09) TxG (*p* = 0.225, η^2^_p_ = 0.05)
Grip Strength (kg)	33.7 (10.4)	35.1 (12.9)	1.4 (5.1)	34.2 (9.3)	34.6 (10.0)	0.4 (3.8)	34.5 (7.7)	37.7 (8.3)	3.2 (3.5)	T (*p* = 0.005, η^2^_p_ = 0.14) G (*p* = 0.827, η^2^_p_ < 0.01) TxG (*p* > 0.130, η^2^_p_ = 0.07)
Row (max reps)	29.7 (8.7)	37.8 (16.1)	8.1 (13.6)	29.2 (9.2)	31.0 (11.2)	1.8 (7.1)	29.6 (14.2)	37.2 (20.8)	7.5 (10.6)	T (*p* < 0.001, η^2^_p_ = 0.23) G (*p* = 0.631, η^2^_p_ = 0.02) TxG (*p* = 0.150, η^2^_p_ = 0.07)
Chest Press (max reps)	31.1 (16.1)	36.6 (17.1)	5.5 (6.2)	28.5 (9.6)	37.4 (18.2)	8.9 (13.6)	27.3 (10.6)	35.9 (16.2)	8.6 (10.1)	T (*p* = 0.001, η^2^_p_ = 0.37) G (*p* = 0.882, η^2^_p_ < 0.01) TxG (*p* = 0.683, η^2^_p_ = 0.02)
Shoulder Press (max reps)	18.5 (8.6)	27.4 (13.0)	8.9 (7.7)	18.2 (6.1)	23.7 (9.7)	5.5 (5.9)	18.6 (9.3)	27.0 (13.1)	8.4 (7.7)	T (*p* = 0.001, η^2^_p_ = 0.54) G (*p* = 0.772, η^2^_p_ < 0.01) TxG (*p* = 0.303, η^2^_p_ = 0.04)
RDL (max reps)	32.1 (12.9)	39.6 (19.9)	7.5 (15.9)	26.0 (11.8)	31.6 (14.4)	5.6 (11.4)	27.5 (7.9)	42.0 (15.1)	14.5 (11.0)	T (*p* < 0.001, η^2^_p_ = 0.35) G (*p* = 0.202, η^2^_p_ = 0.06) TxG (*p* = 0.097, η^2^_p_ = 0.08)
Goblet Squat (max reps)	32.0 (13.4)	43.9 (19.2)	11.9 (12.5)	33.7 (24.0)	42.9 (28.5)	9.3 (15.4)	35.9 (12.9)	54.4 (23.5)	18.5 (14.3)	T (*p* < 0.001, η^2^_p_ = 0.49) G (*p* = 0.453, η^2^_p_ = 0.03) TxG (*p* = 0.127, η^2^_p_ = 0.08)
Hollow Hold (seconds)	70.9 (32.1)	90.8 (52.9)	19.8 (33.0)	93.7 (61.2)	103.2 (78.5)	9.4 (32.4)	90.8 (56.1)	114.4 (63.5)	23.6 (25.5)	T (*p* < 0.001, η^2^_p_ = 0.26) G (*p* = 0.479, η^2^_p_ = 0.03) TxG (*p* = 0.350, η^2^_p_ = 0.04)

Values are presented in mean (standard deviation). Romanian deadlift (RDL). Kilogram (kg). Maximum repetitions (max reps). Time (T). Group (G). Interaction (TxG).

## Data Availability

The data presented in this study are available on request from the corresponding author due to privacy restrictions.

## Supplementary Materials

The following supporting information can be downloaded at: https://www.mdpi.com/article/10.3390/jfmk11020240/s1. References in supplementary Materials can be found [44,45,46,47,48].
