# Supplementary material for: How Much Is Enough: A Randomized Non-Inferiority Trial Comparing Three Bodyweight Training Protocols
Source: J Funct Morphol Kinesiol. 2026 Jun 17;11(2):240. doi: 10.3390/jfmk11020240 (PMC13301418; doi:10.3390/jfmk11020240)
Supplement: Supplementary file 1 [file jfmk-11-00240-s001.zip › Supplemental File S1.pdf]

Supplementary Document

A: Reducing Bias

Reducing Selection Bias

Selection bias results from improper randomization of participants and/or a lack of allocation concealment (Higgins et al. 2011). P.M. performed all participant screening, baseline, and post-intervention testing. Following the provision of informed consent, P.M. assigned each participant with a participant ID code. Participants were randomized following the completion of baseline testing using stratified block randomization and blinded third-party allocation, to ensure equal dispersion of sex and baseline fitness across groups and to protect allocation concealment. Males and females were divided into two blocks based on their respective cardiorespiratory fitness (CRF) at baseline. Participants were divided into an above-average (>50mL/Kg/min for males, >45mL/Kg/min for females) or below-average (<50mL/Kg/min for males, <45mL/Kg/min for females) block. Within each of the four blocks, participants were randomized into 1 of 3 bodyweight training (BWT) protocols by a blinded third-party (K.S.) using a random sequence generator.

Reducing Performance Bias

Performance bias occurs when unblinded investigators' and/or participants' knowledge of group/sequence assignment influences the implementation of an intervention (Higgins et al. 2011). Due to the nature of the study, performance bias was a concern because participants and investigators who supervised the exercise sessions could not be blinded to the assigned intervention. P.M. was present at all baseline and experimental visits and provided equal levels of verbal encouragement to all participants during each bout of exercise to mitigate performance

bias. Further, participants remained uninformed of the study hypothesis until completion of the study.

Reducing Detection Bias

Detection bias occurs as a result of systematic differences in how outcome assessment is performed between groups (Mansournia et al. 2017). Statistical analyses were performed by a second investigator (B.G.) who was not present for any testing and blinded to participant group allocation. The CONSORT statement for non-pharmacological trials states that blinding outcome assessors effectively reduces sources of potential observer bias (Boutron et al. 2017).

Reducing Attrition Bias

Attrition bias occurs due to systematic differences between groups in the number of participants analyzed or excluded for each outcome (Nunan et al. 2018). To reduce this form of bias, a flow chart of participant screening, inclusion and exclusion provided the number of participants (n) and the reason they were included or excluded from each outcome assessment. N is reported in all figure captions to further ensure transparency and mitigate attrition bias.

Reducing Outcome Reporting Bias

Outcome reporting bias involves authors only reporting results that confirm their *a priori* hypothesis (Dwan et al. 2013). To reduce this form of bias, the proposed trial was registered through Open Science Framework (OSF registration), and the primary outcome in the registry matched the outcome presented in the final manuscript.

B: Prior Elicitation Details

Expert elicitation was used to construct informative priors for the Bayesian analyses. Elicitation was applied to reference means for the TAB group and to pairwise treatment differences for 5BX–TAB and AMRAP–TAB. For each outcome, experts provided a median and interquartile range (IQR) for each quantity. These judgements were represented as Normal prior distributions, with the elicited median used as the prior location and the elicited IQR converted to a standard deviation using

$$s \approx \frac{Q_{75} - Q_{25}}{1.349},$$

where 1.349 is the expected IQR of a standard Normal distribution.

Beliefs about individual-level variability were elicited as a plausible range covering most individuals. Where experts supplied a central 95% range $[L, U]$, this was translated to a residual standard deviation using

$$\sigma \approx \frac{U - L}{4}.$$

To ensure positivity and allow uncertainty in residual variation, priors for residual standard deviations were specified on the log scale and back-transformed for model fitting.

For each outcome j, parameter k, and expert e, the elicited prior was written as

$$\pi_{ejk}(\theta_{jk}) = N(m_{ejk}, s_{ejk}^2),$$

where m_{ejk} and s_{ejk}^2 denote the elicited mean and variance. The four expert priors were combined within each outcome and parameter using equal-weight logarithmic pooling:

$$\pi_{jk}(\theta_{jk}) \propto \prod_{e=1}^{4} \pi_{ejk}\left(\theta_{jk}\right)^{w_e}, \qquad w_e = 1/4.$$

Because all expert priors were Normal, the pooled prior was also Normal:

$$\theta_{jk} \sim N\left(\hat{\theta}_{jk}, \hat{v}_{jk}\right),$$

with pooled precision and mean given by

$$\hat{v}_{jk}^{-1} = \sum_{e=1}^{4} \frac{w_e}{s_{ejk}^2}, \qquad \hat{\theta}_{jk} = \hat{v}_{jk} \sum_{e=1}^{4} \frac{w_e m_{ejk}}{s_{ejk}^2}.$$

The same pooling approach was used for residual standard deviations after first transforming expert judgements to the log scale.

To define population-level priors for the hierarchical model, the pooled outcome-specific estimates were synthesised across outcomes. The pooled means $\hat{\theta}_{jk}$, with corresponding variances $\hat{v}_{jk}$, were treated as inputs to a random-effects meta-analysis across outcomes to estimate a domain-level mean and standard error for each parameter class. This produced hyper-prior estimates for the TAB reference mean, denoted $N\left(\mu_{\text{dom}}, SE_{\mu}^2\right)$, and for the treatment differences 5BX–TAB and AMRAP–TAB, denoted $N\left(\delta_{\text{dom},5BX}, SE_{5BX}^2\right)$ and $N\left(\delta_{\text{dom},AMRAP}, SE_{AMRAP}^2\right)$, respectively. These hyper-prior distributions were then used as the population-level priors in the Bayesian model:

$$\alpha_{\text{TAB}} \sim N\left(\mu_{\text{dom}}, SE_{\mu}^2\right),$$

$$\beta_{5BX} \sim N\left(\delta_{\text{dom},5BX}, SE_{5BX}^2\right), \qquad \beta_{AMRAP} \sim N\left(\delta_{\text{dom},AMRAP}, SE_{AMRAP}^2\right).$$

Here, α_{TAB} denotes the population-level reference mean for TAB, while β_{5BX} and β_{AMRAP} denote the population-level effects for 5BX and AMRAP relative to TAB. This procedure makes explicit how expert-level priors were converted into pooled outcome-level priors and then into population-level priors for the hierarchical Bayesian model.

C: Characterization of BWT Protocols

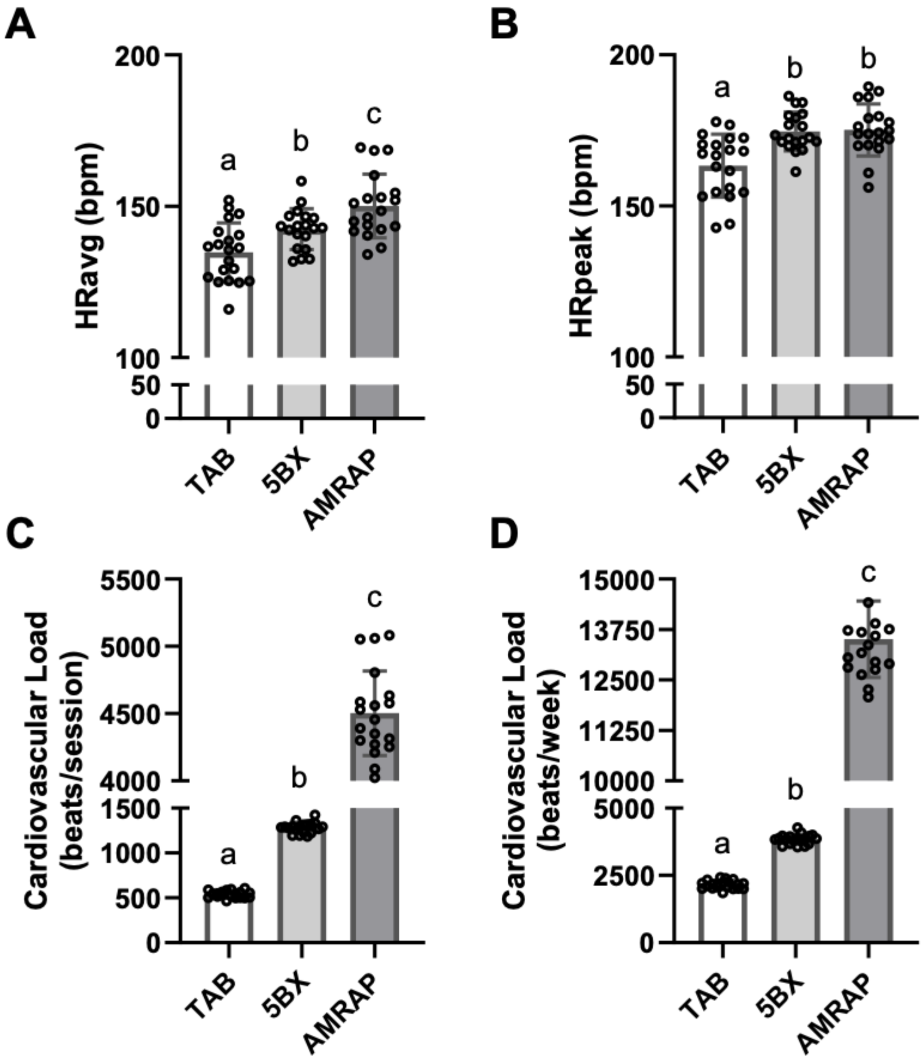

Figure S1. A Average heart rate, **B** Peak heart rate, **C** Sessional cardiovascular load, and **D** Weekly cardiovascular load for TAB, 5BX and AMRAP. Different letters indicate statistically significant differences between groups.

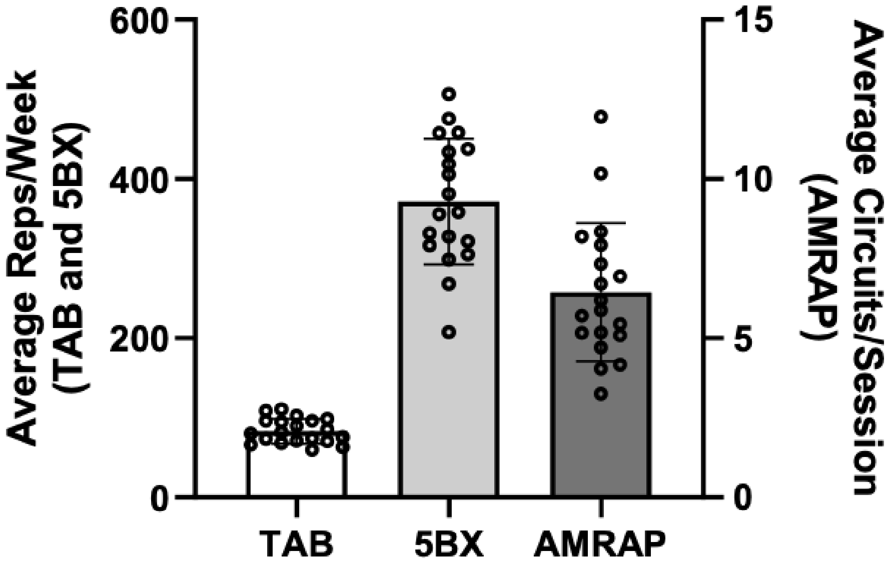

Figure S2. Average repetitions per week (TAB and 5BX) and circuits per session (AMRAP).

D: 2-way ANOVA Analysis of Absolute WRpeak

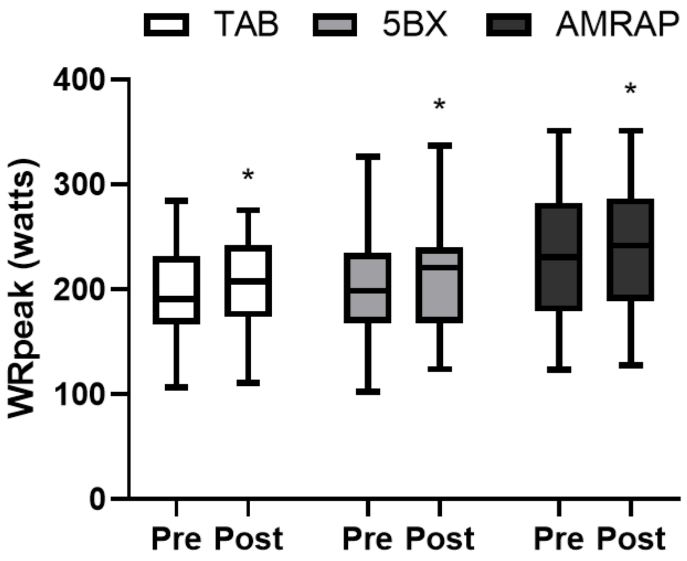

Figure S3. Average pre- and post-training absolute WRpeak values by group. *Denotes a significant main effect of time.

E: Exploratory Analysis of the Effect of Sex on Changes in eVO_2peak.

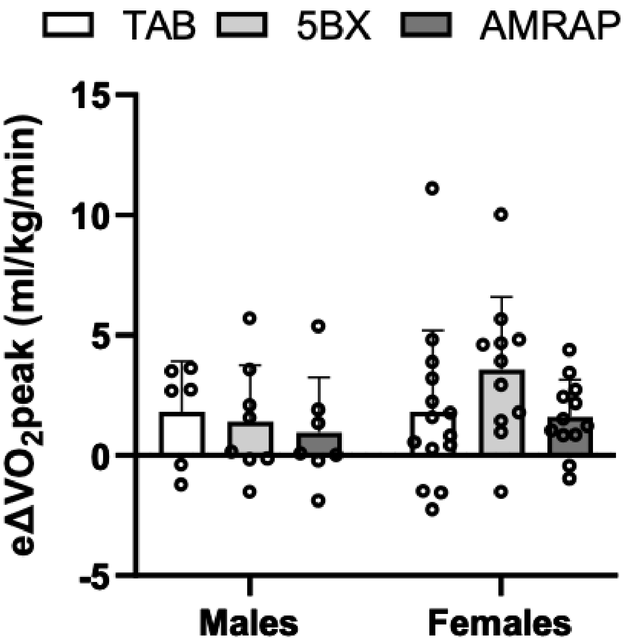

Figure S4. Exploratory sex analysis examining differences in training response for eVO2peak across groups and between sexes.

F: Full Bayesian Results.

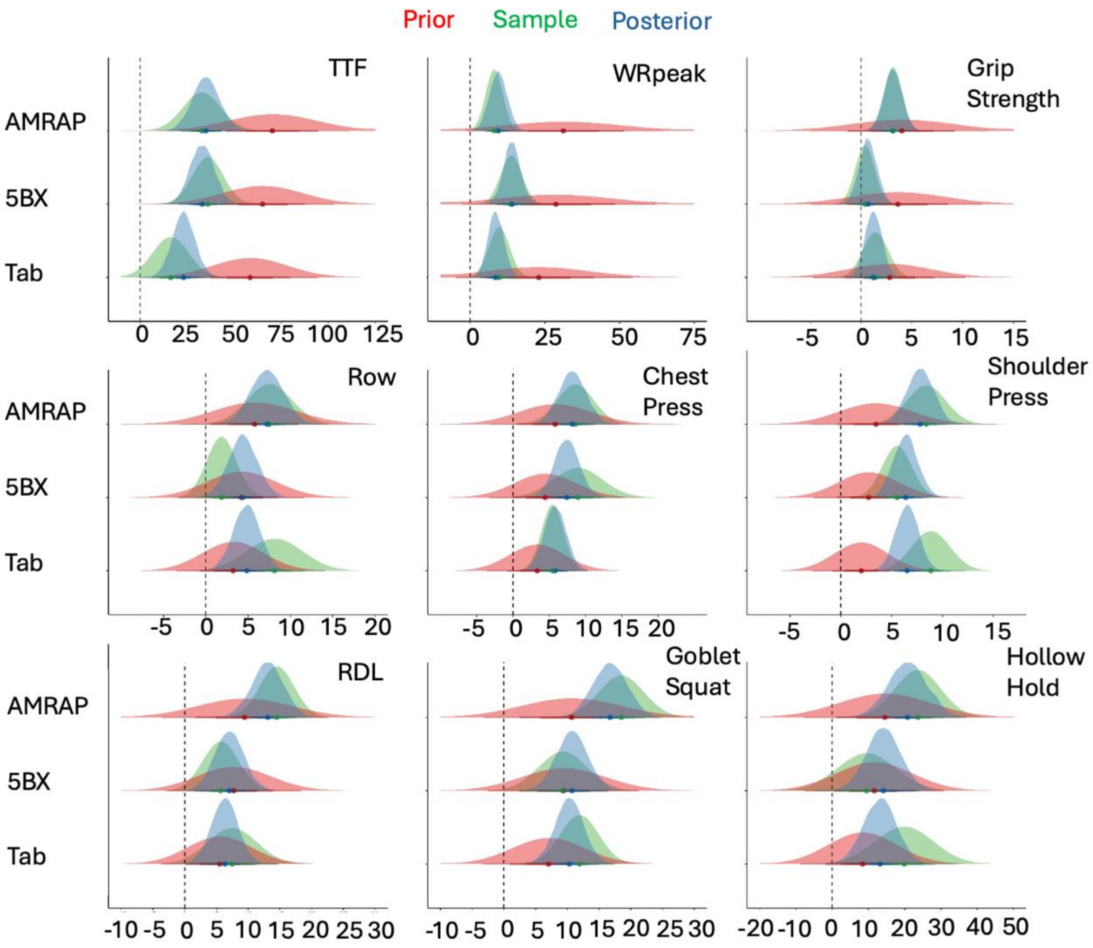

Figure S5. Curves show the probability distributions for change scores by group for each source: prior (elicited via quartiles and pooled across experts) sample (observed data), and posterior (prior + data under the hierarchical model). Points with horizontal bars mark posterior means with 95% credible intervals.

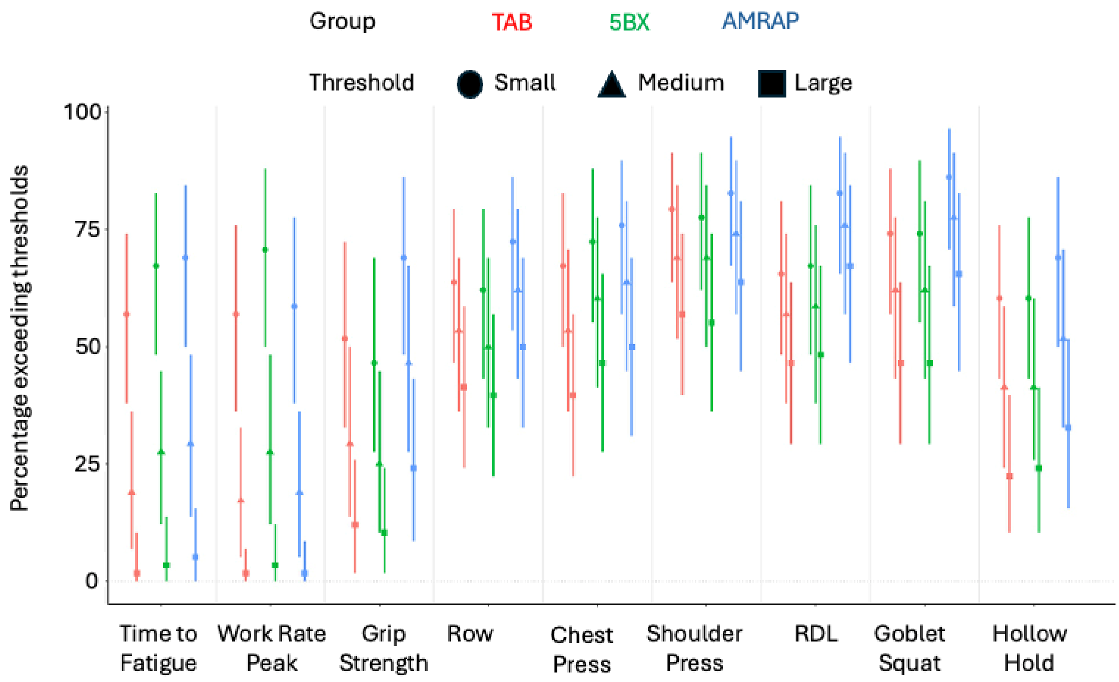

Figure S6. Responder rates by effect-size threshold (small / medium / large) expected across interventions and outcomes. Symbols are posterior point estimates of the percentage of positive responders. Lines represent 95% credible intervals (CrI).

References

Boutron, I., Altman, D.G., Moher, D., Schulz, K.F., Ravaud, P., and for the CONSORT NPT Group. 2017. CONSORT Statement for Randomized Trials of Nonpharmacologic Treatments: A 2017 Update and a CONSORT Extension for Nonpharmacologic Trial Abstracts. Ann Intern Med **167**(1): 40. doi:10.7326/M17-0046.

Dwan, K., Gamble, C., Williamson, P.R., Kirkham, J.J., and the Reporting Bias Group. 2013. Systematic Review of the Empirical Evidence of Study Publication Bias and Outcome Reporting Bias — An Updated Review. PLoS ONE **8**(7): e66844. doi:10.1371/journal.pone.0066844.

Higgins, J.P.T., Altman, D.G., Gotzsche, P.C., Juni, P., Moher, D., Oxman, A.D., Savovic, J., Schulz, K.F., Weeks, L., and Sterne, J.A.C. 2011. The Cochrane Collaboration's tool for assessing risk of bias in randomised trials. BMJ **343**(oct18 2): d5928–d5928. doi:10.1136/bmj.d5928.

Mansournia, M.A., Higgins, J.P.T., Sterne, J.A.C., and Hernán, M.A. 2017. Biases in Randomized Trials: A Conversation Between Trialists and Epidemiologists. Epidemiology **28**(1): 54–59. doi:10.1097/EDE.0000000000000564.

Nunan, D., Aronson, J., and Bankhead, C. 2018. Catalogue of bias: attrition bias. BMJ EBM **23**(1): 21–22. doi:10.1136/ebmed-2017-110883.
